# Editorial: External factors influencing stem cells’ pluripotency, senescence, and differentiation

**DOI:** 10.3389/fcell.2025.1733564

**Published:** 2025-11-07

**Authors:** Mustapha Najimi, Myon Hee Lee

**Affiliations:** 1 Laboratory of Pediatric Hepatology and Cell Therapy, Institute of Experimental and Clinical Research-UCLouvain, Brussels, Belgium; 2 Laboratory of Cell Fate Reprogramming, Hematology/Oncology Division, Department of Medicine, Brody School of Medicine at East Carolina University, Greenville, NC, United States

**Keywords:** stem cells, pluripotency, senescence, differentiation, microenvironment, bioactivemolecules, reprogramming, regenerative medicine

## Introduction

Stem cells have the unique capacities for self-renewal and differentiation into multiple types of functional cells, which are critical for regenerative medicine and tissue engineering (Ireland and Simmons, 2015). Their fate, such as whether they self-renew, differentiate, or enter senescence, is tightly regulated not only by intrinsic genetic and epigenetic factors but also by a complex array of extrinsic factors. In particular, external factors, including the microenvironment, receptor-ligand interactions, and mechanical forces, play critical roles in maintaining stem cell pluripotency, directing differentiation, and preventing or inducing senescence (Zhang et al., 2022; Sun et al.). Aberrant regulation of these extrinsic factors often leads to the loss of normal stem cells and other functional cells, or promotes the formation of unwanted cells, such as cancer stem cells or malignant cancer cells (Ponomarev et al., 2022).

This Research Topic compiles the latest research on external factors that regulate stem cell pluripotency, senescence, and differentiation. It emphasizes that understanding the complex regulation of these extrinsic factors is crucial for optimizing stem cell therapies and preventing abnormalities like cancer by integrating recent research, innovative methodologies, mechanistic insights, and future therapeutic strategies to improve outcomes.

## Regulation of microenvironment, metabolism, and senescence by external factors

Several studies focus on how the cellular environment and metabolic factors govern stem cell senescence. Sun et al. emphasize the role of the external microenvironment cues, such as extracellular matrix (ECM) components, mechanical stimuli, and oxidative stress, in regulating mesenchymal stem cell (MSC) senescence. They underscore that the aging process can be modulated by altering these external factors. Zheng et al. focus on how advanced glycation and products (AGEs), known to accumulate in diabetic and aged tissues, contribute to accelerated senescence and reduced differentiation potential through oxidative damage and inflammatory pathways. Using *Drosophila* as a model, Yan et al. demonstrate the potential of anti-diabetic drugs like dapagliflozin to mitigate intestinal stem cell aging through downregulation of MAPK signaling, suggesting pharmacological avenues for rejuvenating aged stem cells. Huang et al. explore the regenerative effects of MSC-conditioned medium (MSC-CM) in a diabetic wound model. They show that MSC-CM can promote tissue regeneration by influencing cytokine and chemokine signaling pathways, illustrating how paracrine signals serve as powerful external factors. All these findings underscore the intricate interplay between the metabolic conditions, pharmacological agents, and inflammatory signals in modulating stem cell longevity and functionality.

## Differentiation pathways shaped by microenvironment and signaling

Another group of studies emphasizes how external cues direct stem cell differentiation toward specific lineages. Zhang et al. investigate the effect of the preconditioning p38 MAPK pathway on synovium-derived stem cells undergoing chondrogenesis. The study reveals that this preconditioning produces divergent outcomes depending on the ECM conditions, reflecting the context-dependent nature of signal interpretation. Nagalingam et al. reveal key pathways affected during the transition of human induced pluripotent stem cell (iPSC)-derived cardiac fibroblasts to myofibroblasts as demonstrated by using integrated transcriptomic and metabolomic profiling. Khaveh et al. identify key driver genes that mediate the phenotypic stability and differentiation of porcine MSCs, emphasizing the complex interplay between external signals, cell-matrix interactions, and lineage commitment. These studies collectively highlight the critical role of the external signals, in guiding stem cell differentiation.

## Epigenetic and ligand-mediated modulation of stem cell fate

Recent work underscores the potential of small molecules, ligands, and epigenetic modifiers to influence stem cell fate and plasticity. Bae et al. provide an in-depth review of histone modification and its role in maintaining pluripotency and reprogramming in stem cells, with potential applications for cancer stem cell therapy. The authors propose that extrinsic signals can dynamically alter chromatin states, thereby modulating stem cell identity. Brown investigates retinoic acid receptor (RAR) signaling and its regulatory role in cell fate decision-making, suggesting that exogenous retinoic acid acts as a powerful switch in stem cell differentiation. Liu et al. expand their landscape by introducing ginsenosides, bioactive compounds from ginseng, as natural modulators capable of guiding stem cell behavior. These studies collectively point to the promising application of external small molecules and epigenetic modifiers to precisely reprogram stem cell identities.

## Integrative perspective and clinical implications

Overall, the authors also emphasize the clinical challenges related to genetic instability, reprogramming fidelity, and safety in therapeutic applications. Collectively, this body of work reveals a complex but increasingly decipherable framework of how external signals integrate with intrinsic cellular networks to regulate stem cell behavior. Despite these hurdles, the growing understanding of this intricate framework offers promising avenues for advancing regenerative therapies and disease modeling by enabling precise modulation of stem cell functions.

## Conclusion

This Research Topic collectively advances our understanding of how external factors shape stem cell identity and fate. From metabolic stressors and inflammatory cytokines to mechanical forces and small-molecule ligands, the external environment plays an indispensable role in directing stem cell responses. In conclusion, controlling stem cell fate is not solely an intrinsic endeavor and requires a deep understanding of how the external environment communicates with stem cells to modulate their identity, potential, and regenerative capabilities.
